# A representation learning model based on variational inference and graph autoencoder for predicting lncRNA-disease associations

**DOI:** 10.1186/s12859-021-04073-z

**Published:** 2021-03-21

**Authors:** Zhuangwei Shi, Han Zhang, Chen Jin, Xiongwen Quan, Yanbin Yin

**Affiliations:** 1 College of Artificial Intelligence, Nankai University, Tongyan Road, 300350 Tianjin, China; 2 College of Computer Science, Nankai University, Tongyan Road, 300350 Tianjin, China; 3 Department of Food Science and Technology, Nebraska Food for Health Center, University of Nebraska-Lincoln, 1400 R Street, Lincoln, NE 68588 USA

**Keywords:** Variational inference, Graph autoencoder, lncRNA-disease association, Representation learning

## Abstract

**Background:**

Numerous studies have demonstrated that long non-coding RNAs are related to plenty of human diseases. Therefore, it is crucial to predict potential lncRNA-disease associations for disease prognosis, diagnosis and therapy. Dozens of machine learning and deep learning algorithms have been adopted to this problem, yet it is still challenging to learn efficient low-dimensional representations from high-dimensional features of lncRNAs and diseases to predict unknown lncRNA-disease associations accurately.

**Results:**

We proposed an end-to-end model, VGAELDA, which integrates variational inference and graph autoencoders for lncRNA-disease associations prediction. VGAELDA contains two kinds of graph autoencoders. Variational graph autoencoders (VGAE) infer representations from features of lncRNAs and diseases respectively, while graph autoencoders propagate labels via known lncRNA-disease associations. These two kinds of autoencoders are trained alternately by adopting variational expectation maximization algorithm. The integration of both the VGAE for graph representation learning, and the alternate training via variational inference, strengthens the capability of VGAELDA to capture efficient low-dimensional representations from high-dimensional features, and hence promotes the robustness and preciseness for predicting unknown lncRNA-disease associations. Further analysis illuminates that the designed co-training framework of lncRNA and disease for VGAELDA solves a geometric matrix completion problem for capturing efficient low-dimensional representations via a deep learning approach.

**Conclusion:**

Cross validations and numerical experiments illustrate that VGAELDA outperforms the current state-of-the-art methods in lncRNA-disease association prediction. Case studies indicate that VGAELDA is capable of detecting potential lncRNA-disease associations. The source code and data are available at https://github.com/zhanglabNKU/VGAELDA.

**Supplementary Information:**

The online version contains supplementary material available at 10.1186/s12859-021-04073-z.

## Introduction

LncRNAs are RNAs longer than 200 nucleotides thus losing the function of encoding, while they can still influence a series of biological processes, such as gene transcription, cell apoptosis, hormonal regulation, and immune response. Hence, lncRNAs are closely linked to plenty of human diseases [1–3]. For instance, lncRNA PANDAR is a novel biomarker of breast cancer, which upregulates proliferation of breast cancer cells [4]. Sun et al. [5] found that the downregulation of lncRNA MEG3 promotes proliferation of gastric cancer cells. Faghihi et al. [6] reported that lncRNA BACE1-AS can regulate mRNA BACE1, while BACE1 is associated with the generation of beta-amyloid, which can cause Alzheimer’s disease. Therefore, it is essential to predict potential lncRNA-disease associations for disease prevention, detection, diagnosis and treatment. However, there are only a small number of lncRNA-disease associations that have been discovered so far, and it would be ideal to predict more potential lncRNA-disease associations using computational approaches. Generally, computational methods, especially machine learning algorithms, are more time-efficient and cost-effective to detect potential lncRNA-disease associations compared with experimental methods.

Previous machine learning approaches for predicting lncRNA-disease associations can be categorized into three types. The first type of methods is based on matrix analysis. Two commonly used matrix analysis methods for predicting lncRNA-disease associations are manifold regularization [7] and matrix completion [8], which suggest that lncRNA-disease association matrix follow manifold constraint or low-rank constraint, respectively. Manifold regularization based methods have been widely adopted for link prediction of biological entities [9–11]. Laplacian regularized least square (LRLS) method [7] integrates manifold regularization and basic least square method. Chen and Yan [12] proposed LRLSLDA that applied LRLS to the lncRNA-disease associations prediction, after the construction of an lncRNA graph and a disease graph through computing feature similarity respectively. Based on LRLSLDA, several methods were proposed to improve the performance of LRLS by integrating different types of feature similarities [13, 14]. In addition, lncRNA-disease associations can be viewed as links on an lncRNA-disease bipartite graph. Matrix completion algorithm [8] can solve link prediction problem by applying low-rank constraint to association matrix, and have been commonly applied to forecast associations among biological entities [15–17]. Lu et al. [18] proposed a matrix completion based method for predicting lncRNA-disease associations. Geometric matrix completion [19, 20] incorporates manifold regularization into the matrix completion problem, and Lu et al. [21] proposed a geometric matrix completion based framework for predicting lncRNA-disease associations.

The second type of methods focuses on the integration of heterogeneous features. Applying multi-source features to learn better representations is an efficient technique for predicting associations among biological entities [22, 23]. Lan et al. [24] developed a web server for lncRNA-disease association prediction by integrating multiple features of lncRNAs and diseases to construct lncRNA similarity network and disease similarity network. Fu et al. [25] integrated heterogeneous data for lncRNA-disease associations prediction by matrix factorization with low-rank constraint. Ding et al. [26] inferred links on lncRNA-disease bipartite graph via lncRNA-disease-gene tripartite graph. Yao et al. [27] adopted random forest for feature selection in lncRNA-disease associations prediction.

The third type is deep learning approaches. Neural networks are competent to capture efficient low-dimensional representations from high-dimensional features of biological entities, and deep learning based methods were proposed for detecting potentional associations among biological entities [17, 22, 28]. Thus, several deep learning models applying autoencoders for representation learning of lncRNA features and disease features were proposed [29, 30]. Graph neural networks (GNN) [31] were proposed in deep learning on graphs. Hence, there are some recent approaches for lncRNA-disease associations prediction based on GNN. Xuan et al. [32] integrated graph convolutional networks (GCN) [33] and CNN to learn representations from features of lncRNAs and diseases. GCN is applicable for link prediction on bipartite graph [34], and Wu et al. [35] adopted graph autoencoder to predict lncRNA-disease associations on lncRNA-disease bipartite graph.

In this paper, we proposed a method, VGAELDA, that integrates variational inference and graph autoencoders to improve the performance of lncRNA-disease associations prediction. In previous works, feature inference and label propagation are two separated stages in these methods, and hence label propagation procedure may fail to make the full use of low-dimensional representations learned from high-dimensional features. Using deep learning approaches, our method proposed an end-to-end framework, which fuses feature inference and label propagation under the variational inference algorithm of Graph Markov Neural Networks (GMNN) [36]. Specifically, the feature inference network in VGAELDA is designed as a variational graph autoencoder (VGAE) [37] that learns representations from feature matrices of lncRNAs and diseases respectively. Furthermore, the label propagation network in our model is a graph autoencoder (GAE) [37] that estimates the score of unknown lncRNA-disease pairs from known ones. These two graph autoencoders learn from feature and propagate label alternately, which are trained by variational EM algorithm, and are implemented as a representation learning framework. This framework minimizes the difference of the representations learned by two autoencoders respectively. Therefore, VGAELDA has the following advantages. (i) VGAE is preferable to infer low-dimensional representations from high-dimensional features in a graph, and these representations can better depict similarities and dependencies among nodes. This would significantly enhance the robustness and preciseness of prediction without handcrafted feature similarities. (ii) VGAELDA implements the variational EM algorithm as a representation learning framework, by training the feature inference autoencoder and the label propagation autoencoder alternately. (iii) VGAELDA provides a useful solution to the geometric matrix completion problem via deep learning, because autoencoders tend to minimize the rank of outputs, and we suggest that manifold regularization can be obtained via the alternate training of two graph autoencoders. (iv) VGAELDA implements an efficient way to integrate information from lncRNA space and disease space. Experiments illustrate that VGAELDA is superior to the current state-of-the-art methods, and case studies on several diseases illustrate the capability of VGAELDA to detect new lncRNA-disease associations.

## Results

### Datasets

In this paper, we adopted two datasets for evaluation. Dataset1 is an lncRNA-disease association dataset from [26], including 540 associations among 115 lncRNAs and 178 diseases. Dataset2 is an lncRNA-disease association dataset from [25], including 2697 associations among 240 lncRNAs and 412 diseases. Both of them were collected from LncRNADisease [38] Database.

For each lncRNA, we adopted Word2Vec to compute the feature vector. Word2Vec [39] is an efficient method to learn the embedding vectors of natural language, and BioVec [40] (https://pypi.org/project/biovec/) applied Word2Vec for representation learning of biological sequences, including protein sequences or nucleotide sequences. In VGAELDA, the length of each vector was set at 300. We downloaded lncRNA sequences from the Nucleotide Database of NCBI.

For each disease, we adopted its associations with 1415 genes as the feature vector on Dataset1. Dataset2 includes disease associated with 15527 genes. After removing genes that are not associated with any diseases, 10146 genes remain and are used as the feature vector on Dataset2. Information with respect to diseases was collected from DisGeNet [41] and Disease Ontology [42].

### Comparison with other methods

**Fig. 1:**
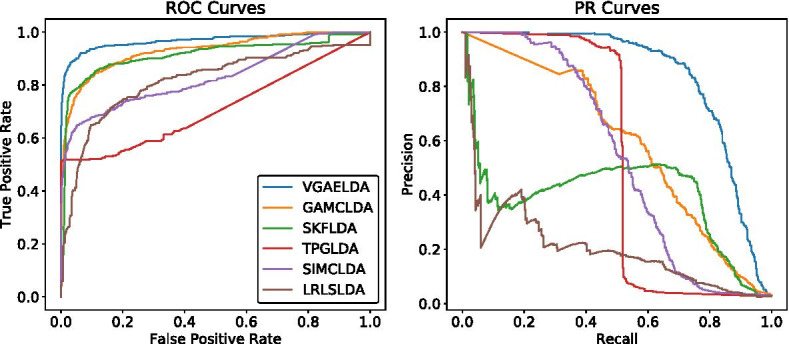
ROC and PR curves of different methods on Dataset1. In AUROC, VGAELDA (AUROC = 0.9680) outperforms GAMCLDA (0.9299), SKFLDA (0.9154), TPGLDA (0.7936), SIMCLDA (0.8293) and LRLSLDA (0.8157). In AUPR, VGAELDA (AUPR = 0.8380) outperforms GAMCLDA (0.5794), SKFLDA (0.4024), TPGLDA (0.5308), SIMCLDA (0.5357) and LRLSLDA (0.2035)

**Fig. 2:**
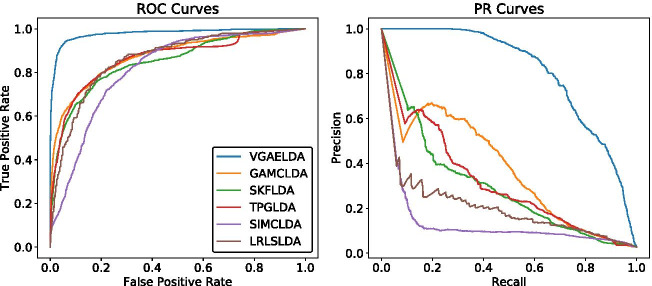
ROC and PR curves of different methods on Dataset2. In AUROC, VGAELDA (AUROC = 0.9692) outperforms GAMCLDA (0.8841), SKFLDA (0.8524), TPGLDA (0.8771), SIMCLDA (0.8146) and LRLSLDA (0.8627). In AUPR, VGAELDA (AUPR = 0.8203) outperforms GAMCLDA (0.3798), SKFLDA (0.2831), TPGLDA (0.3192), SIMCLDA (0.1189) and LRLSLDA (0.1812)

#### Cross validation

We compared our proposed method, VGAELDA, with other five state-of-the-art methods:

- LRLSLDA: Chen and Yan [12] proposed a Laplacian regularized least square (LRLS) method [7] based framework to predict lncRNA-disease associations.
- SIMCLDA: Lu et al. [18] proposed a computational method for predicting lncRNA-disease associations based on speedup inductive matrix completion (SIMC) [43].
- TPGLDA: Ding et al. [26] integrated heterogeneous features by constructing lncRNA-disease-gene tripartite graph for lncRNA-disease associations prediction.
- SKFLDA: Xie et al. [14] proposed SKFLDA that applied kernel fusion trick for different types of similarities to improve the preciseness of lncRNA-disease associations prediction.
- GAMCLDA: Wu et al. [35] implemented GAMCLDA, adopting graph autoencoders to predict lncRNA-disease associations on lncRNA-disease bipartite graph.

We adopted 5-fold cross validation to obtain the result, and the metrics were listed below.

1 $$\begin{aligned} Sensitivity= & {} \frac{TP}{TP+FN}=TPR=Recall, \end{aligned}$$

2 $$\begin{aligned} Specificity= & {} \frac{TN}{TN+FP}=1-FPR, \end{aligned}$$

3 $$\begin{aligned} Accuracy= & {} \frac{TN+TP}{TN+TP+FN+FP}, \end{aligned}$$

4 $$\begin{aligned} Precision= & {} \frac{TP}{TP+FP}, \end{aligned}$$

5 $$\begin{aligned} F1= & {} \frac{2\times Precision\times Recall}{Precision + Recall}, \end{aligned}$$

6 $$\begin{aligned} Mcc= & {} \frac{TP \times TN-FP \times FN}{\sqrt{(TP+FN) \times (TP+FP) \times (TN+FN) \times (TN+FP)}}, \end{aligned}$$

where TP denotes true positive, FN denotes false negative, TN denotes true negative, FP denotes false negative, TPR denotes true positive rate, FPR denotes false positive rate, and Mcc denotes Matthews correlation coefficient. The receiver operating characteristic (ROC) curve can be plotted by TPR and FPR, while the area under ROC curve (AUROC) and the area under precision-recall curve (AUPR) are important metrics to measure the performance of a binary classification model.

We plotted the ROC curves and PR curves of Dataset1 and Dataset2 on Figs. 1 and 2, respectively. We ran our experiments for 5 times, and the mean values and standard deviations of AUROC and AUPR are listed on Table 1. The AUROC and AUPR values of VGAELDA in 5 times are listed in Additional file 1.

The results show that VGAELDA outperforms the other five state-of-the-art methods in both AUROC and AUPR, on both datasets. Specifically, for the AUPR values obtained by other five state-of-the-art methods, GAMCLDA performs best in 5-fold CV on both Dataset1 and Dataset2, which gives AUPR values at 0.5794 and 0.3798 respectively. Compared with these AUPR values, VGAELDA significantly outperforms these previous methods by increasing the AUPR values 45% in 5-fold CV on Dataset1, and 116% in 5-fold CV on Dataset2.

**Table 1:** Mean values and standard deviations of AUROC and AUPR on Dataset1 and Dataset2, compared with different methods

Method	Dataset1		Dataset2	
	AUROC	AUPR	AUROC	AUPR
LRLSLDA	0.8157 ± 0.0005	0.2035 ± 0.0001	0.8627 ± 0.0017	0.1812 ± 0.0021
SIMCLDA	0.8293 ± 0.0023	0.5357 ± 0.0011	0.8146 ± 0.0042	0.1189 ± 0.0076
TPGLDA	0.7936 ± 0.0054	0.5308 ± 0.0028	0.8771 ± 0.0053	0.3192 ± 0.0058
SKFLDA	0.9154 ± 0.0013	0.4024 ± 0.0017	0.8524 ± 0.0066	0.2831 ± 0.0085
GAMCLDA	0.9299 ± 0.0033	0.5794 ± 0.0143	0.8841 ± 0.0110	0.3798 ± 0.0154
VGAELDA	**0.9680** ± 0.0042	**0.8380** ± 0.0041	**0.9692** ± 0.0080	**0.8203** ± 0.0139

The bold number is the highest value of each column, which is achieved by our method, VGAELDA. The bold clarifies the superiority of our method

#### Evaluation on imbalanced data

As the datasets are imbalanced, i.e., the number of negative samples is far more than positive samples, it is essential to evaluate the capability to retrieve true positive samples from predicted positive ones. In our experiments, the evaluation was implemented through the following two ways. In summary, VGAELDA performs the best in both evaluation ways.

Firstly, we evaluated the performance of our model at high stringency level of specificity according to Eq. (2,3,4,5,6). We fixed specificity at 0.95 and 0.99, and then computed sensitivity, accuracy, precision, F1-score and Mcc. The results of Dataset1 and Dataset2 are listed on Additional file 2 and Table 2, respectively, which illustrate that VGAELDA outperforms other five methods at all five metrics, and in both datasets. Matthews correlation coefficient (Mcc) is a comprehensive metric in binary classification on imbalanced data [44]. For the Mcc values obtained by the other five state-of-the-art methods, SKFLDA performs the best at $Sp=0.95$ on Dataset1, which obtains 0.4637, GAMCLDA performs the best at $Sp=0.99$ on Dataset1 and both $Sp=0.95$ and 0.99 on Dataset2, which obtains 0.5804, 0.3855 and 0.4860 respectively. VGAELDA outperforms these methods by improving the Mcc values 13% and 28% at $Sp=0.95$ and 0.99 on Dataset1, and 42% and 49% at $Sp=0.95$ and 0.99 on Dataset2.

**Fig. 3:**
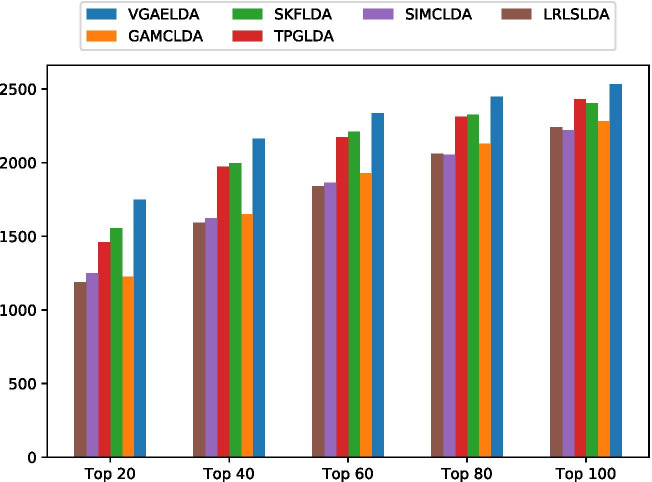
True positive samples at different cutoffs on Dataset2

Secondly, we evaluated recall score (i.e. sensitivity) via counting the number of true positive samples at different top-*k* cutoffs, according to Eq. (1), where $k\in \{20,40,60,80,100\}$. The bar charts depicting the number of true positive samples at different top-*k* cutoffs on Dataset1 and Dataset2 are shown on Additional file 3 and Fig. 3, respectively. VGAELDA retrieves the most true positive samples at all 5 cutoffs on both Dataset1 and Dataset2.

**Table 2:** Binary classification metrics of different methods on Dataset2

Sp	Method	Sn	Acc	Pre	F1	Mcc
0.95	LRLSLDA	0.4572	0.9369	0.2051	0.2831	0.2777
	SIMCLDA	0.2128	0.9299	0.1066	0.1421	0.1169
	TPGLDA	0.5565	0.9394	0.2384	0.3338	0.3380
	SKFLDA	0.5284	0.9385	0.2286	0.3191	0.3206
	GAMCLDA	0.6377	0.9415	0.2635	0.3729	0.3855
	VGAELDA	**0.9329**	**0.9495**	**0.3434**	**0.5020**	**0.5490**
0.99	LRLSLDA	0.1591	0.9676	0.3145	0.2113	0.2086
	SIMCLDA	0.1020	0.9658	0.2223	0.1398	0.1348
	TPGLDA	0.2673	0.9703	0.4279	0.3291	0.3238
	SKFLDA	0.2354	0.9694	0.3976	0.2958	0.2913
	GAMCLDA	0.4472	0.9752	0.5558	0.4956	0.4860
	VGAELDA	**0.7831**	**0.9843**	**0.6868**	**0.7318**	**0.7254**

The bold number is the highest value of each column, which is achieved by our method, VGAELDA. The bold clarifies the superiority of our method

*Sp* specificity, *Sn* sensitivity, *Acc* accuracy, *Pre* precision, *F1* F1-score, *Mcc* Matthews correlation coefficient

### Case studies

To further evaluate the capability for detecting unknown lncRNA-disease associations of VGAELDA, case studies were adopted. We predicted the unknown disease-related lncRNAs of some specific diseases on the datasets, which can be validated by PubMed literature. The unknown disease-related lncRNAs of a disease are ranked by VGAELDA-predicted score. In this paper, we adopted case studies on lncRNAs associated with breast cancer and colon cancer.

On Dataset 1, the top 10 VGAELDA-predicted lncRNAs associated with breast cancer and colon cancer were listed in Tables 3 and 4, respectively. PMID denotes the PubMed ID of the supporting literature for the corresponding disease-related lncRNAs detected by VGAELDA. Table 3 indicates that all the top 10 VGAELDA-predicted lncRNAs associated with breast cancer have been confirmed by previous literature. Table 4 indicates that 8 of the top 10 VGAELDA-predicted lncRNAs associated with colon cancer have been confirmed as well.

On Dataset 2, the top 10 VGAELDA-predicted lncRNAs associated with breast cancer and colon cancer were listed in Additional files 4 and 5. Additional file 4 demonstrates that 8 of the top 10 VGAELDA-predicted lncRNAs associated with breast cancer have been confirmed by previous literature. Additional file 5demonstrates that 9 of the top 10 VGAELDA-predicted lncRNAs associated with colon cancer have been confirmed.

Breast cancer is the most commonly diagnosed cancer and the main threat of health among females worldwide [45]. VGAELDA has been applied to predict potential lncRNAs related to breast cancer. For instance, DNM3OS downregulates Vitamin D receptor (VDR), and VDR is capable of upregulating Suppressor of fused gene (SuFu), while SuFu is an inhibitor of progression of breast cancer [46]. CCAT1 promotes proliferation and migration of triple-negative breast cancer cells via downregulating miRNA miR-218 and activating the expression of protein ZFX [47]. BANCR is significantly correlated to the growth of breast cancer cells [48].

Colon cancer is a major malignant cancer in digestive system [45]. Among the top 10 lncRNAs predicted by VGAELDA, UCA1 facilitates the progression of colon cancer through upregulating miRNA miR-28-5p and HOXB3 [49]. It is found that GAS5 is positively correlated to colon cancer as well [50]. Also, previous research suggests that PVT1 can sponge miRNA miR-26b and promote proliferation and metastasis of colon cancer [51].

Besides, we listed the predictions of potential lncRNA-disease associations with respect to all diseases of Dataset1 and Dataset2 in Additional files 6 and 7, respectively.

**Table 3:** Top 10 predicted lncRNAs associated with breast cancer on Dataset1

Rank	lncRNA name	PMID
1	DNM3OS	27693451
2	CCAT1	31310241
3	BANCR	29565494
4	PANDAR	26927017
5	MNX1-AS1	30697072
6	FOXCUT	25516208
7	WRAP53	26460974
8	TUG1	30098551
9	MIR17HG	25680407
10	IGF2-AS	33175607

**Table 4:** Top 10 predicted lncRNAs associated with colon cancer on Dataset1

Rank	lncRNA name	PMID
1	UCA1	30652355
2	GAS5	27951730
3	PVT1	30504754
4	SNHG16	31502038
5	XIST	29679755
6	DNM3OS	Unconfirmed
7	TUG1	27634385
8	IGF2-AS	28534511
9	HULC	30551459
10	SPRY4-IT1	Unconfirmed

## Discussion

Previous methods for predicting lncRNA-disease associations modeled dependent relationship from features based on some handcrafted measurements of similarity, then propagated labels of samples on the graph constructed via feature similarities. However, it is difficult for those measurements to capture similarities among high-dimensional features directly. Hence, the hyperparameters in these measurements would significantly affect the performance of prediction, which decreases the preciseness of label propagation.

To address this issue, VGAELDA designed representation learning framework that fuses the feature inference network and the label propagation network, to solve graph semi-supervised learning Problem 1 (see Methods). Our Assumption 1 (see Methods) clarifies the capability of an autoencoder to obtain low-rank solution. Based on Assumption 1, an autoencoder with manifold loss as we defined in Definition 1 (see Methods), is competent to obtain the optimal solution of geometric matrix completion problem. Considering the manifold constraint and low-rank constraint that the lncRNA-disease association matrix should satisfy, we adopted VGAE to implement feature inference network GNNq, and GAE to implement label propagation network GNNp. With the alternate training via variational EM algorithm, two GAEs with manifold loss to measure the smoothness of manifold, would significantly strengthen the robustness and preciseness of label propagation through the representations learned by VGAE. Hence the feature similarities, i.e. the topological relationship of the graph, only need to be estimated roughly. The experiments demonstrate that VGAELDA outperforms various kinds of matrix completion based or manifold regularization based methods.

Furthermore, VGAELDA provides an efficient way to integrate information from lncRNA space and disease space. By applying co-training loss as we defined in Definition 2 (see Methods), information from lncRNA space and disease space are captured collaboratively. Finally, the association matrix $F_l$ computed from lncRNA space and $F_d$ computed from disease space, can be integrated simply, since Assumption 1 suggest that both $F_l$ and $F_d$ follow low-rank property.

## Conclusion

The prediction of potential lncRNA-disease associations is of great importance to disease prognosis, diagnosis and treatment. In this paper, we proposed a deep learning model, VGAELDA, which integrates variational inference and graph autoencoders to detect potential lncRNA-disease associations. VGAELDA designed a representation learning framework to fuse the feature inference network and the label propagation network. Specifically, VGAELDA adopts variational graph autoencoder GNNq for feature inference, and graph autoencoder GNNp for label propagation. These two graph autoencoders are trained alternately in end-to-end manner via variational EM algorithm. This has significantly improved the efficiency of feature representation learning and label propagation. Further discussion demonstrates the validity of VGAELDA to find an optimal solution to the geometric matrix completion problem, and to integrate information from both lncRNA space and disease space. Experiments illustrate that VGAELDA is superior to the current state-of-the-art prediction methods, and case studies indicate that VGAELDA is competent in detecting potential lncRNA-disease associations. The results of evaluation demonstrate that VGAELDA is competent to capture efficient low-dimensional representations from high-dimensional features of both lncRNAs and diseases, and predict unknown lncRNA-disease associations robustly and precisely.

Compared to previous lncRNA-disease associations prediction methods, VGAELDA adopts an end-to-end framework based on variational inference in graph neural networks. VGAELDA is a data-driven end-to-end deep learning approach with a high flexibility. Therefore, VGAELDA is competent to be a general model for graph semi-supervised learning and association prediction tasks for other biological entities.

## Methods

### Problem formulation

Suppose the number of lncRNAs and diseases are *m* and *n* respectively, and $Y_{m\times n}$ denotes the association matrix. $Y_{ij}=1$ if the association between lncRNA *i* and disease *j* is known, otherwise $Y_{ij}=0$. An algorithm predicting lncRNA-disease associations requires *Y* and corresponding feature matrix *X* as input, then outputs a score for each pair of lncRNA and disease. *F* denotes the score matrix, $F_{ij}\in [0,1]$, i.e. the prediction result.

In the view of machine learning, an lncRNA-disease pair is labeled if it has been proved to be associated. Usually, there are only few samples labeled in an lncRNA-disease dataset, and the other tremendous amount of associations need to be detected. Therefore, the prediction for lncRNA-disease associations can be viewed as propagating labels to plenty of unlabeled pairs from few labeled ones, which is classified as semi-supervised learning.

### Variational inference for graph semi-supervised learning

#### Graph semi-supervised learning

Semi-supervised learning is based on manifold assumption [52]. Manifold assumption clarifies that samples are distributed on a manifold, samples with higher feature similarities are closer on the manifold, and tend to share the same labels. The manifold of data can be depicted by graph structure constructed through feature matrix, which leads to graph semi-supervised learning. This type of methods first computes adjacency matrix from features to construct a graph, then propagate labels from labeled samples to unlabeled ones on this graph iteratively [53, 54].

Suppose *L* denotes normalized Laplacian matrix of the graph, minimizing $\mathrm {trace}(F^TLF)$ can obtain the label matrix *F* following manifold assumption [52, 55]. Belkin et al. [7] added this manifold constraint to least square problem, then derived Laplacian regularized least square (LRLS) method

7 $$\begin{aligned} \min _F \,\, \Vert F-Y\Vert _F^2+\eta \mathrm {trace}(F^TLF), \end{aligned}$$

where $\Vert \cdot \Vert _F$ denotes Frobenius norm of a matrix, and $\eta$ is a hyperparameter. Eq. (7) is a trade-off between the accuracy based on labeled data, and the smoothness of the manifold. This is classified as manifold regularization [7]. Label propagation follows the framework of manifold regularization as Eq. (7) [53, 54]. Xia et al. [9] derived that association matrix *F* follows manifold assumption, and can be obtained via solving Eq. (7).

**Fig. 4:**
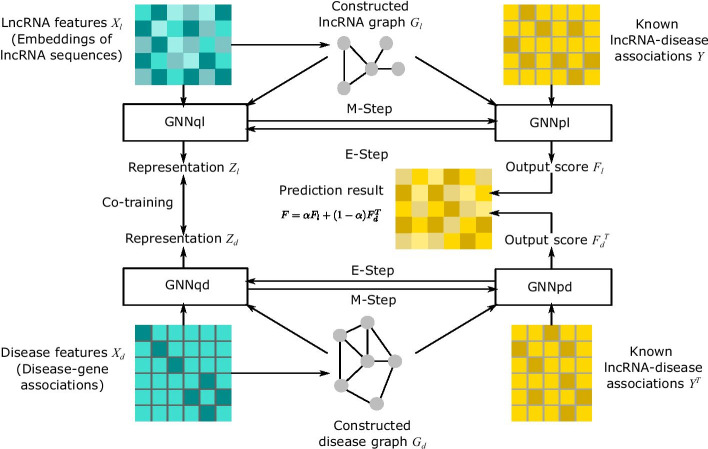
Framework of VGAELDA. Step 1: lncRNA features $X_l$ are embeddings of lncRNA sequences computed by Word2Vec, while disease features $X_d$ are associations with genes. Step 2: constructing graph $G_l$ and $G_d$ through Eq. (16) for lncRNAs and diseases, respectively. Step 3: GNNql and GNNpl are applied to $G_l$, that they require $X_l$ and *Y* as inputs, while GNNqd and GNNpd applied to $G_d$ require $X_d$ and $Y^T$ as inputs. Step 4: training GNNq and GNNp alternately via variational EM algorithm, while training GNNql and GNNqd collaboratively. Step 5: final result fusion by Eq. (28)

#### Graph Markov neural networks

The motivation of VGAELDA is begun with graph semi-supervised learning from probabilistic perspective. Through this perspective, label propagation can be viewed as maximizing $p(y_u|y_l,x_v)$ [56], where $y_u$ and $y_l$ denote labels from unlabeled and labeled nodes respectively, and $x_v$ denotes attributes of objects on the graph. As the number of $y_u$ is often much larger than $y_l$, it is difficult to maximize $p(y_u|y_l,x_v)$. Qu et al. [36] proposed Graph Markov Neural Networks (GMNN), suggesting that variational inference for graph semi-supervised learning leads to Problem 1.

##### Problem 1

*Variational inference for graph semi-supervised learning adopts the variational distribution* $q(y_u|x_v)$ *to approximate* $p(y_u|y_l,x_v)$, *which leads to optimize evidence lower bound* (ELBO)

8 $$\begin{aligned} {\mathbb {E}}_{q(y_u|x_v)}[\log q(y_u|x_v)-\log p(y_l,y_u|x_v)]. \end{aligned}$$

Remark of Problem 1 is in the Additional file 8. Since labeled and unlabeled samples are observations and latent variables in conditional random field (CRF), and according to Markov property in CRF, the label of an unlabeled node is only related to its neighborhood. Hence, label propagation procedure aggregates messages from neighborhood, which is intrinsically related to graph neural networks [33].

GMNN adopted two GNNs, GNNq and GNNp, to depict $q(y_u|x_v)$ and $p(y_l,y_u|x_v)$ respectively, since GNNs are successfully adopted in graph semi-supervised learning [33]. Problem 1 can be solved by variational EM (expectation maximization) algorithm [57] (see Additional file 8), GNNq and GNNp are trained by variational EM algorithm, which executes the following two steps alternately until convergence.

- E-step: fix GNNp, and train GNNq by attributes of objects, to obtain the pseudo-labels,
- M-step: fix GNNq, and input pseudo-labels into GNNp for training.

### Geometric matrix completion

Except for manifold assumption, the association matrix also follows the low-rank assumption that it lies in a smaller subspace, this leads to the matrix completion [8] problem.

9 $$\begin{aligned} \min _F\,\, \mathrm {rank}(F)\quad \mathrm {s.t.}\,\, {\mathcal {P}}_\Omega (F)={\mathcal {P}}_\Omega (Y), \end{aligned}$$

where $\Omega$ is the set of all known lncRNA-disease associations. The projection operator ${\mathcal {P}}_\Omega (\cdot ):{\mathbb {R}}^{m\times n}\rightarrow {\mathbb {R}}^{m\times n}$ of matrix *M* is defined as

10 $$\begin{aligned} {\mathcal {P}}_\Omega (M)_{ij}={\left\{ \begin{array}{ll} M_{ij} &{} (i,j)\in \Omega \\ 0 &{} \mathrm {otherwise} \end{array}\right. }. \end{aligned}$$

Eq. (9) is an NP-hard and nonconvex problem, thus it is usually relaxed as the following convex surrogate

11 $$\begin{aligned} \min _F\,\, \Vert F\Vert _*+\mu \Vert {\mathcal {P}}_\Omega (F-Y)\Vert _F^2. \end{aligned}$$

where $\Vert \cdot \Vert _*$ denotes nuclear norm, i.e. the sum of singular values of a matrix.

Geometric matrix completion [19, 20] incorporates manifold constraint $\mathrm {trace}(F^TLF)$ into low-rank constraint, that is to solve

12 $$\begin{aligned} \min _F\,\,\Vert F\Vert _*+\mu \Vert {\mathcal {P}}_\Omega (F-Y)\Vert _F^2+\eta \mathrm {trace}(F^TLF). \end{aligned}$$

### VGAELDA

#### Method overview

We proposed our model, VGAELDA, which designed representation learning framework to fuse the feature inference network and the label propagation network, and is trained through variational EM algorithm using GMNN [36] that integrated variational inference and GNN. VGAELDA executes the following two steps alternately until convergence.

- E-step (feature inference): fix GNNp, and train GNNq by high-dimensional features, to obtain low-dimensional representations,
- M-step (label propagation): fix GNNq, and input lncRNA-disease association matrix into GNNp for training.

In VGAELDA, feature inference network GNNq is a variational graph autoencoder (VGAE) [37], and label propagation network GNNp is a graph autoencoder (GAE) [37]. Assumption 1 and Definition 1 suggest that the application of these two autoencoders solves the geometric matrix completion problem Eq. (12), for capturing efficient low-dimensional representations via VGAELDA. Furthermore, VGAELDA adopts co-training [58] that integrates information from lncRNA space and disease space. The framework of our model is shown on Fig. 4.

#### Implementing graph autoencoders

Each layer of a graph autoencoder is graph convolutional layer. The formula of the *l*-th $(l>0)$ graph convolutional [33] layer is

13 $$\begin{aligned} H^{(l)}=\rho ({\tilde{D}}^{-1/2}{\tilde{A}} \tilde{D}^{-1/2}H^{(l-1)}\Theta ^{(l)}), \end{aligned}$$

where ${\tilde{A}}$ is adjacency matrix with self-loop, i.e. $\tilde{A}=A+I$. ${\tilde{D}}$ is a diagonal matrix called degree matrix, ${\tilde{D}}_{ii}=\sum _j{\tilde{A}}_{ij}$, $\rho (\cdot )$ denotes nonlinear activation function, $\Theta ^{(l)}$ denotes weight of the *l*-th layer of network, and $H^{(0)}$ is the initial input feature matrix.

##### Assumption 1

Autoencoder GNNp with *Y* as input and *F* as output can obtain the optimal solution of Eq. (11).

##### Definition 1

(manifold loss) Suppose *Z* and $Z'$ are representations of autoencoder GNNq and GNNp, respectively, then, to optimize manifold constraint $\mathrm {trace}(F^TLF)$ can be viewed as optimizing the following manifold loss

14 $$\begin{aligned} L_m=\frac{1}{2}\Vert Z-Z'\Vert _F^2. \end{aligned}$$

Remarks of Assumption 1 and Definition 1 are in Additional file 8. In the view of the alternating direction method of multipliers (ADMM) [59], solving the geometric matrix completion problem Eq. (12) can be viewed as optimizing Eq. (7) and Eq. (11) alternately. Therefore, autoencoder GNNp with the addition of manifold loss as we defined in Definition 1, obtains the solution of Eq. (12).

However, to enhance the efficiency of adding manifold loss Eq. (14), we implemented a variational graph autoencoder as GNNq to capture representation *Z*. Suppose the feature matrix of the graph is *X*, the encoder learns mean $\mu$ and standard deviation $\sigma$. The representation *Z* can be computed by applying reparameterization trick [60], which means

15 $$\begin{aligned} Z=\mu +\sigma \epsilon , \end{aligned}$$

where $\epsilon$ is sampled from standard Gaussian distribution. Then, the decoder reconstructs a feature matrix $X'$.

The adjacency matrix of graph *G* can be constructed simply in this way. Firstly, sort the Euclidean distances among different feature vectors of nodes. Secondly, for each node *i*, select the 10-nearest nodes except itself. Thirdly, suppose the set of these nodes for node *i* is ${\mathcal {N}}(i)$, matrix *C* satisfies that $C_{ij}=1$ if $j\in {\mathcal {N}}(i)$, otherwise $C_{ij}=0$. The adjacency matrix with self-loop of the constructed graph *G* is

16 $$\begin{aligned} {\tilde{A}}=C^T\odot C+I, \end{aligned}$$

where $\odot$ denotes Hadamard product.

Network structures of GNNq and GNNp are shown on Additional file 9. As shown on Additional file 9, GNNp is a basic GAE that takes initial label matrix *Y* as input, the dimension of hidden vector is 256, output of hidden layer is $Z'$, and output of decoder is prediction *F*. GNNq is a VGAE, that each layer of the variational autoencoder [60] is a graph convolutional layer, the dimension of output vectors of each hidden layers in GNNq are 256.

#### Variational EM algorithm

The variational EM algorithm is implemented through minimizing the losses of GNNq and GNNp alternately. Similar to other variational graph autoencoders, the loss function of GNNq is the sum of reconstruction error $L_{qr}$, and KL divergence $L_{KL}$.

17 $$\begin{aligned} L_q=L_{qr}+L_{KL}. \end{aligned}$$

Kingma and Welling [60] derived that in a variational autoencoder:

- If the features follow Gaussian distribution, the reconstruction error is mean square error.
  18 $$\begin{aligned} L_{qr}=\frac{1}{2}\Vert X-X'\Vert _F^2, \end{aligned}$$
- If the features follow Bernoulli distribution, the reconstruction error is cross entropy loss.
  19 $$\begin{aligned} L_{qr}=-\sum _{i,j}X_{ij}\log X'_{ij}. \end{aligned}$$
- KL divergence loss can be computed through
  20 $$\begin{aligned} L_{KL}=-\sum _{i,j}\frac{1}{2}(1+2\log \sigma _{ij}-\mu _{ij}^2-\sigma _{ij}^2). \end{aligned}$$

In VGAELDA, the features of lncRNAs are computed from sequences by Word2Vec [39], and features of diseases are computed through associations with disease-related genes. Thus, lncRNA features follow Gaussian distribution, and disease features follow Bernoulli distribution. Therefore, $L_{qr}$ in GNNql and GNNqd are computed by Eq. (18) and Eq. (19), respectively.

The outputs of encoder and decoder are scaled into (0,1) through applying sigmoid activation function. Meanwhile, following Eq. (7) , the loss function of GNNp is the sum of reconstruction error and manifold loss.

21 $$\begin{aligned} L_p=L_{pr}+\gamma L_m. \end{aligned}$$

The reconstruction error of GNNp is the cross entropy between prediction and true label

22 $$\begin{aligned} L_{pr}=-\sum _{i,j}Y_{ij}\log F_{ij}. \end{aligned}$$

Then, *F* is obtained after adopting variational EM algorithm to train GNNq and GNNp alternately until convergence, and is finally scaled into interval [0, 1] by

23 $$\begin{aligned} F_{ij}\leftarrow \frac{F_{ij}-F_{min}}{F_{max}-F_{min}}, \end{aligned}$$

where $F_{min}$ and $F_{max}$ denote minimum and maximum element in matrix *F*.

#### Integrating information from lncRNA space and disease space

As shown on Fig. 4, the constructed lncRNA graph $G_l$ and disease graph $G_d$ are different. Eq. (17) and Eq. (21) can compute loss from $G_l$ and $G_d$ respectively, but it is important to integrate the information capturing from lncRNA space and disease space. Therefore, we adopt co-training [58] to train GNNql and GNNqd collaboratively.

##### Definition 2

(co-training loss) Suppose $Z_l$ and $Z_d$ are representations learned from lncRNA space and disease space, respectively, then co-training loss

24 $$\begin{aligned} L_c=\frac{1}{2}\Vert Z_lZ_d^T-Y\Vert _F^2. \end{aligned}$$

can measure the performance of co-training.

Remark of Definition 2 is in Additional file 8. Then GNNql and GNNqd are trained simultaneously by optimizing the total loss of GNNq

25 $$\begin{aligned} {\mathcal {L}}_q=\alpha L_{ql}+(1-\alpha )L_{qd}+\beta L_c, \end{aligned}$$

where $L_{ql}$ and $L_{qd}$ denote losses of GNNql and GNNqd computed through Eq. (17) respectively, and $\alpha \in (0,1)$ is the weight parameter that balances information capturing from lncRNA space and disease space. Similarly, the total loss of GNNp is

26 $$\begin{aligned} {\mathcal {L}}_p=\alpha L_{pl}+(1-\alpha )L_{pd}, \end{aligned}$$

where $L_{pl}$ and $L_{pd}$ denote losses of GNNpl and GNNpd computed through Eq. (21) respectively. Then, the variational EM algorithm is implemented through optimizing ${\mathcal {L}}_q$ and ${\mathcal {L}}_p$ alternately. After training procedure, GNNpl outputs $F_l$ while GNNpd outputs $F_d$. Since both $F_l\in {\mathbb {R}}^{m\times n}$ and $F_d\in {\mathbb {R}}^{n\times m}$ are low-rank provided by autoencoders, and through the rank-sum inequality that

27 $$\begin{aligned} \mathrm {rank}(aF_l+ bF_d^T) \le \mathrm {rank}(F_l) + \mathrm {rank}(F_d^T),\forall a,b, \end{aligned}$$

the final result

28 $$\begin{aligned} F=\alpha F_l+(1-\alpha )F_d^T. \end{aligned}$$

is low-rank.

The procedure of VGAELDA is summarized in Algorithm 1, where $X',Z\leftarrow \mathrm {GNN}(G,X)$ summarizes the computing procedure of a GAE.

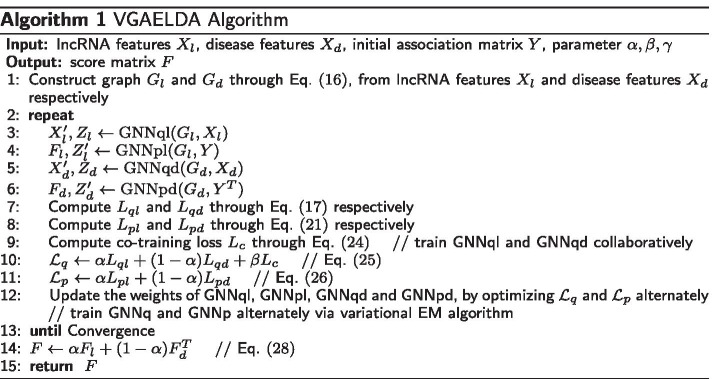



### Hyperparameters tuning

In VGAELDA, there are three hyperparameters, $\alpha ,\beta$ and $\gamma$, that need to be tuned. Hyperparameter $\alpha$ depicts a balance between lncRNA space and disease space. However, after evaluating our model at each $\alpha \in \{0.1,0.3,0.5,0.7,0.9\}$, we found that VGAELDA is robust to the choice of $\alpha$, and the results are shown on Additional file 10. Hence we simply set $\alpha =0.5$.

Since manifold loss $L_m$ and co-training loss $L_c$ depend on the computation of representations of GNNql and GNNqd, the capabilities of manifold constraint and co-training constraint are related to the effectiveness of representation capturing by GNNq. Hence, we need to set hyperparameter $\beta$ in Eq. (25) and $\gamma$ in Eq. (21), increasing as training goes, to enhance the robustness of representation learning, and the convergence of EM algorithm. So here we set $\beta =\gamma =e/e_n$ at *e*-th epoch, where $e_n=500$ denotes the number of epochs.

We adopted PyTorch [61] (https://pytorch.org/) to construct VGAELDA, and applied Adam optimizer [62], where learning rate is 0.01, weight decay is $10^{-5}$, and we set dropout=0.5 [63]. Our model was trained on a single NVIDIA GeForce GTX 2070 GPU with 8GB memory. we evaluated the performance of VGAELDA through varying learning rate in {0.001,0.01,0.1,1}, and the results are shown on Additional file 11. The figure depicts that the best value of learning rate is 0.01.

Moreover, we evaluated our model at different dimension of hidden vectors, and the results are shown on Additional file 12. The figure depicts that the performance of our model is enhanced with the increase of hidden vector dimension. However, when the dimension is more than 256, there is little increment and the performance remains stable. Hence, we set the hidden vector dimension at 256 to save the time and space cost of our model.

Besides, we also evaluated our model at different dimension of lncRNA embedding vectors adopted by Word2Vec, and the results are shown on Additional file 13. The figure shows that a larger dimension of lncRNA embedding vectors tends to perform better. However, when the dimension is more than 150, there is little increment and the performance remains stable. Hence, we simply set the dimension of lncRNA embedding vectors at 300.

## Supplementary Information

**Additional file 1.** AUROC and AUPR values of VGAELDA in 5 times

**Additional file 2.** Binary classification metrics of different methods on Dataset1

**Additional file 3.** True positive samples at different cutoffs on Dataset1

**Additional file 4.** Case study for breast cancer on Dataset2

**Additional file 5.** Case study for colon cancer on Dataset2

**Additional file 6.** Predictions of potential lncRNA-disease association on Dataset1

**Additional file 7.** Predictions of potential lncRNA-disease association on Dataset2

**Additional file 8.** Remarks

**Additional file 9.** Network structures

**Additional file 10.** AUPR at different *α*

**Additional file 11.** AUPR at different learning rate

**Additional file 12.** AUPR at different dimension of hidden vectors

**Additional file 13.** AUPR at different dimension of embedding vectors of lncRNA

## Data Availability

All the data using in our paper are collected from the following public datasets. Dataset1 can be downloaded from https://github.com/USTC-HIlab/TPGLDA. Dataset2 can be downloaded from http://mlda.swu.edu.cn/codes.php?name=MFLDA. Both of them were collected from LncRNADisease Database (http://www.cuilab.cn/lncrnadisease). In VGAELDA, the information of lncRNA sequences was downloaded from the Nucleotide Database of NCBI (https://www.ncbi.nlm.nih.gov/nuccore), and the information of diseases was downloaded from DisGeNet (https://www.disgenet.org/home/) and Disease Ontology (https://disease-ontology.org/). The source code is available at https://github.com/zhanglabNKU/VGAELDA.

## Abbreviations

Acc: Accuracy
AUPR: Area under precision-recall curve
AUROC: Area under ROC curve
EM: Expectation maximization
FN: False negative
FP: False positive
GAE: Graph autoencoder
GCN: Graph convolutional networks
GNN: Graph neural networks
Mcc: Matthews correlation coefficient
LDA: lncRNA-disease association
LncRNA: Long non-encoding RNA
LOOCV: Leave-one-out cross validation
LRLS: Laplacian regularized least square method
Pre: Precision
ROC curve: Receiver operating characteristic (ROC) curve
Sn: Sensitivity
Sp: Speciality
TN: True negative
TP: True positive
VGAE: Variational graph autoencoder
