# Supplementary material for: A representation learning model based on variational inference and graph autoencoder for predicting lncRNA-disease associations
Source: BMC Bioinformatics. 2021 Mar 21;22:136. doi: 10.1186/s12859-021-04073-z (PMC7983260; doi:10.1186/s12859-021-04073-z)
Supplement: Supplementary file 2 — Additional file 2. Binary classification metrics of different methods on Dataset1 [file 12859_2021_4073_MOESM2_ESM.pdf]

Table S2: Binary classification metrics of different methods on Dataset1. Sp denotes specificity. Sn denotes sensitivity. Acc denotes accuracy. Pre denotes precision. F1 denotes F1-score. Mcc denotes Matthews correlation coefficient.

Sp	Method	Sn	Acc	Pre	F1	Mcc
0.95	LRLSLDA	0.4130	0.9355	0.1819	0.2525	0.2449
	SIMCLDA	0.6426	0.9419	0.2582	0.3684	0.3834
	TPGLDA	0.5185	0.9384	0.2188	0.3077	0.3100
	SKFLDA	0.7852	0.9457	0.2986	0.4327	0.4637
	GAMCLDA	0.7593	0.9449	0.2914	0.4212	0.4493
	VGAELDA	**0.9019**	**0.9485**	**0.3273**	**0.4803**	**0.5257**
0.99	LRLSLDA	0.2019	0.9690	0.3494	0.2559	0.2507
	SIMCLDA	0.4926	0.9767	0.5684	0.5278	0.5173
	TPGLDA	0.5167	0.9776	0.5861	0.5492	0.5389
	SKFLDA	0.2611	0.9708	0.4147	0.3205	0.3149
	GAMCLDA	0.5722	0.9791	0.6107	0.5908	0.5804
	VGAELDA	**0.8130**	**0.9853**	**0.6881**	**0.7453**	**0.7406**
