# Supplementary figures and images for: A representation learning model based on variational inference and graph autoencoder for predicting lncRNA-disease associations
Source: BMC Bioinformatics. 2021 Mar 21;22:136. doi: 10.1186/s12859-021-04073-z (PMC7983260; doi:10.1186/s12859-021-04073-z)

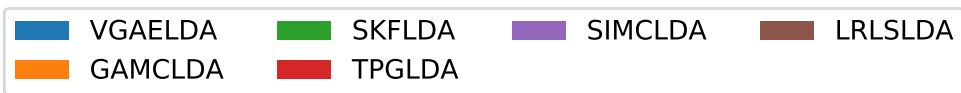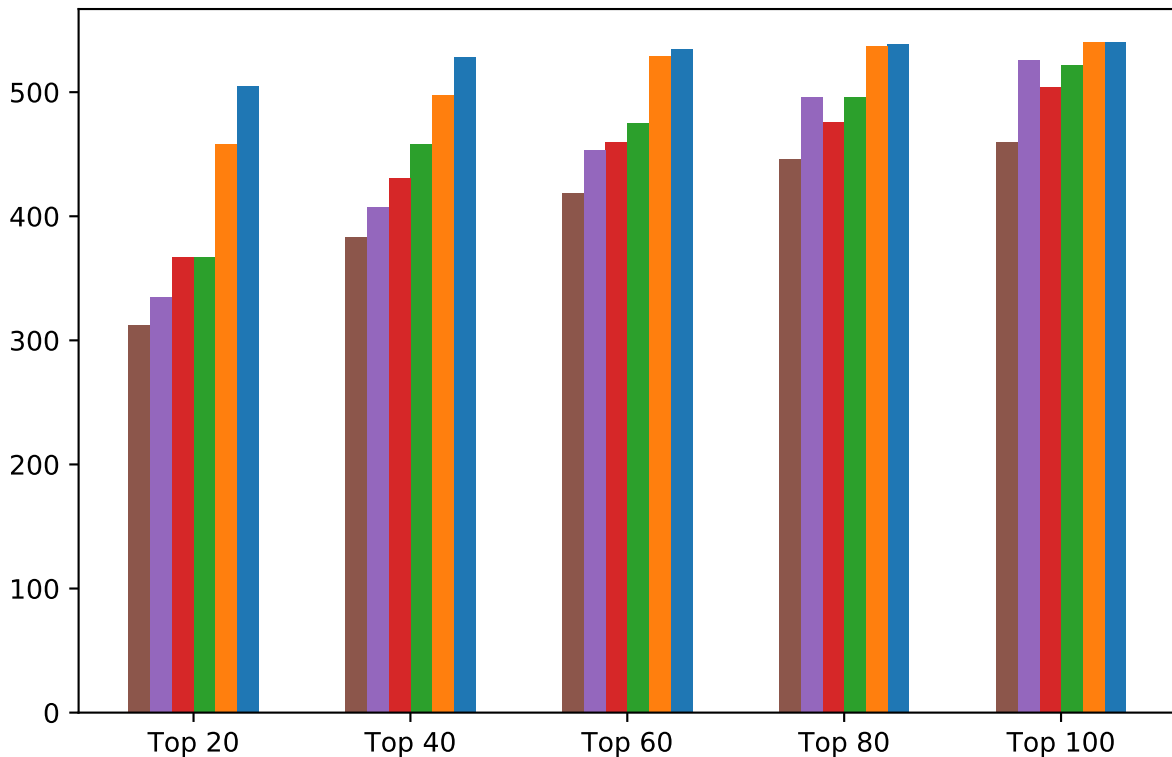

Supplement: Supplementary file 3 — Additional file 3. True positive samples at different cutoffs on Dataset1 [file 12859_2021_4073_MOESM3_ESM.pdf]

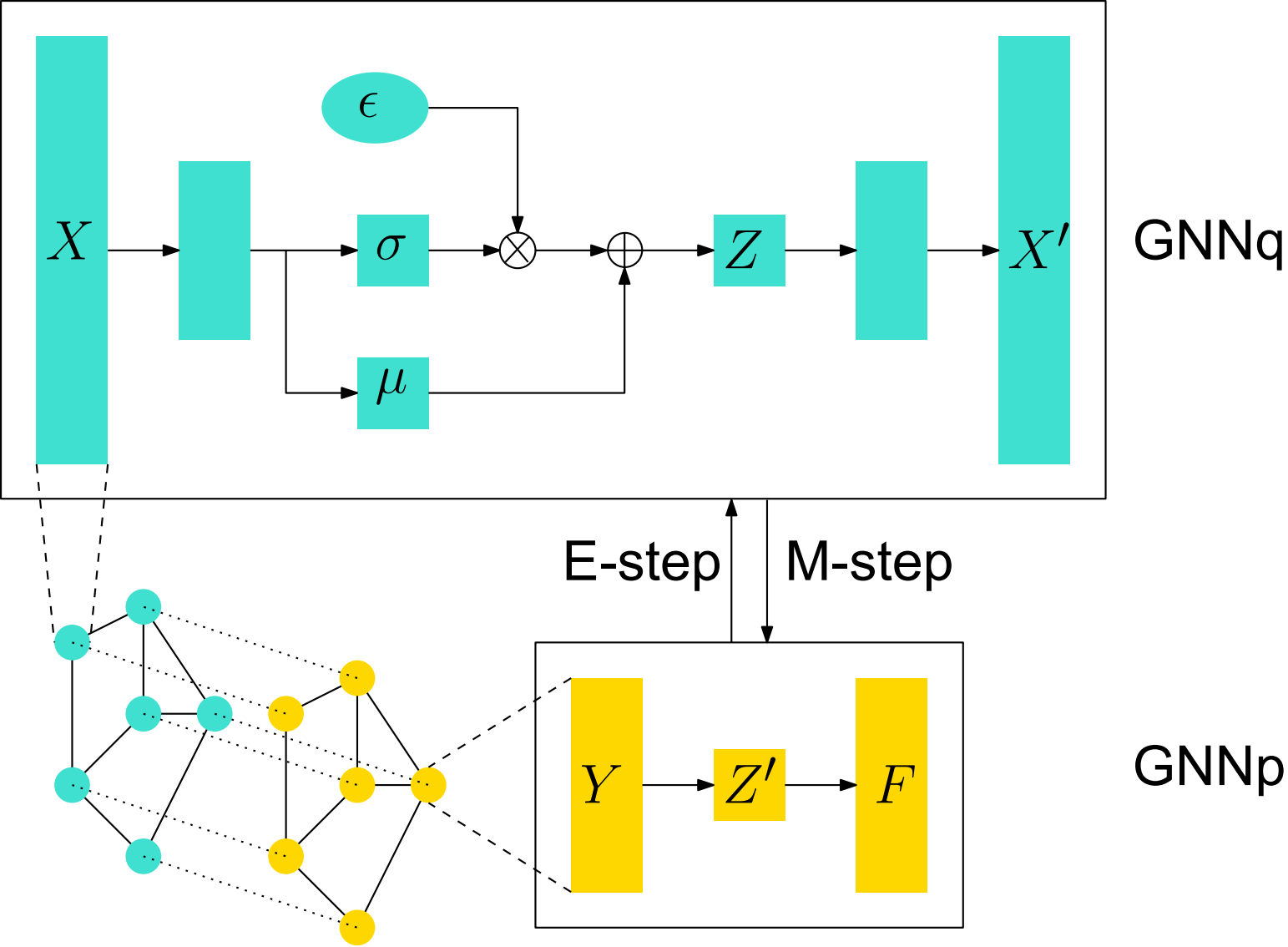

Supplement: Supplementary file 9 — Additional file 9. Network structures [file 12859_2021_4073_MOESM9_ESM.pdf]

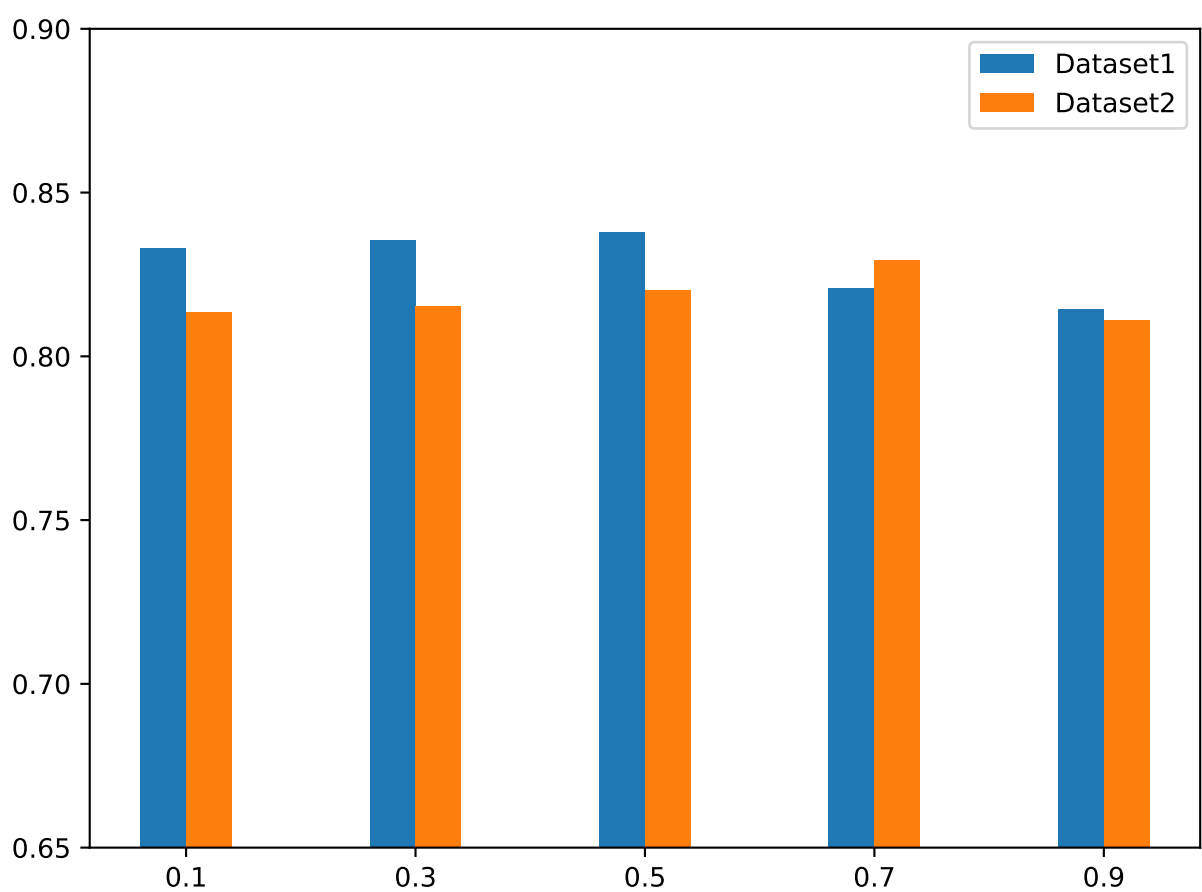

Supplement: Supplementary file 10 — Additional file 10. AUPR at different α [file 12859_2021_4073_MOESM10_ESM.pdf]

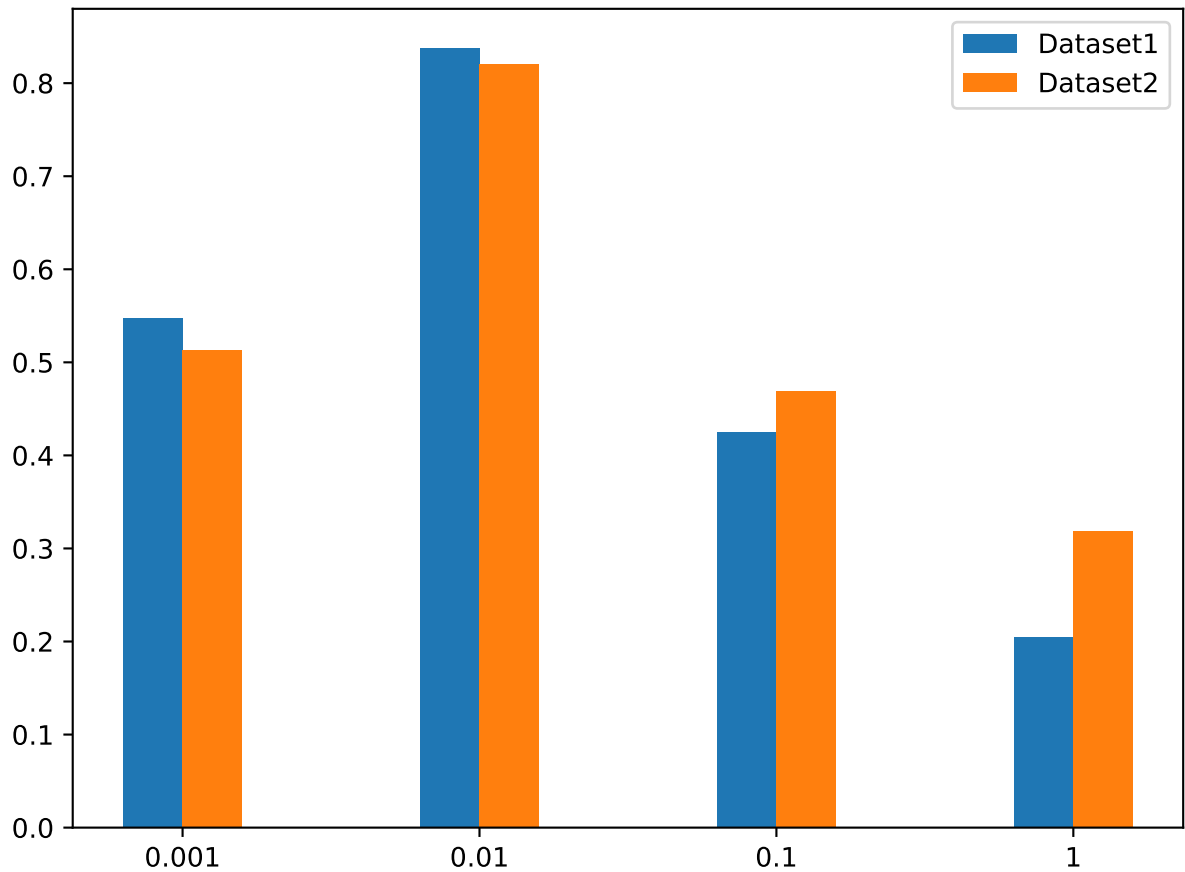

Supplement: Supplementary file 11 — Additional file 11. AUPR at different learning rate [file 12859_2021_4073_MOESM11_ESM.pdf]

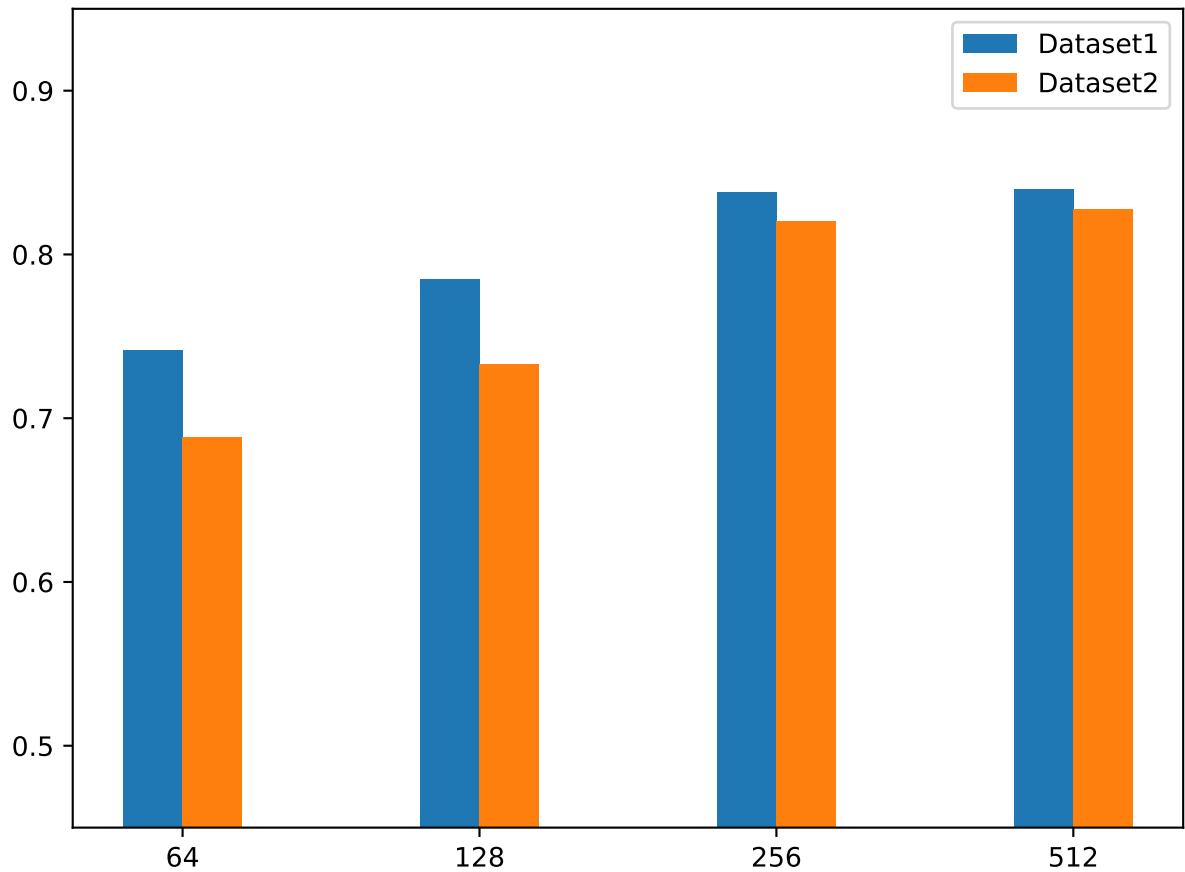

Supplement: Supplementary file 12 — Additional file 12. AUPR at different dimension of hidden vectors [file 12859_2021_4073_MOESM12_ESM.pdf]

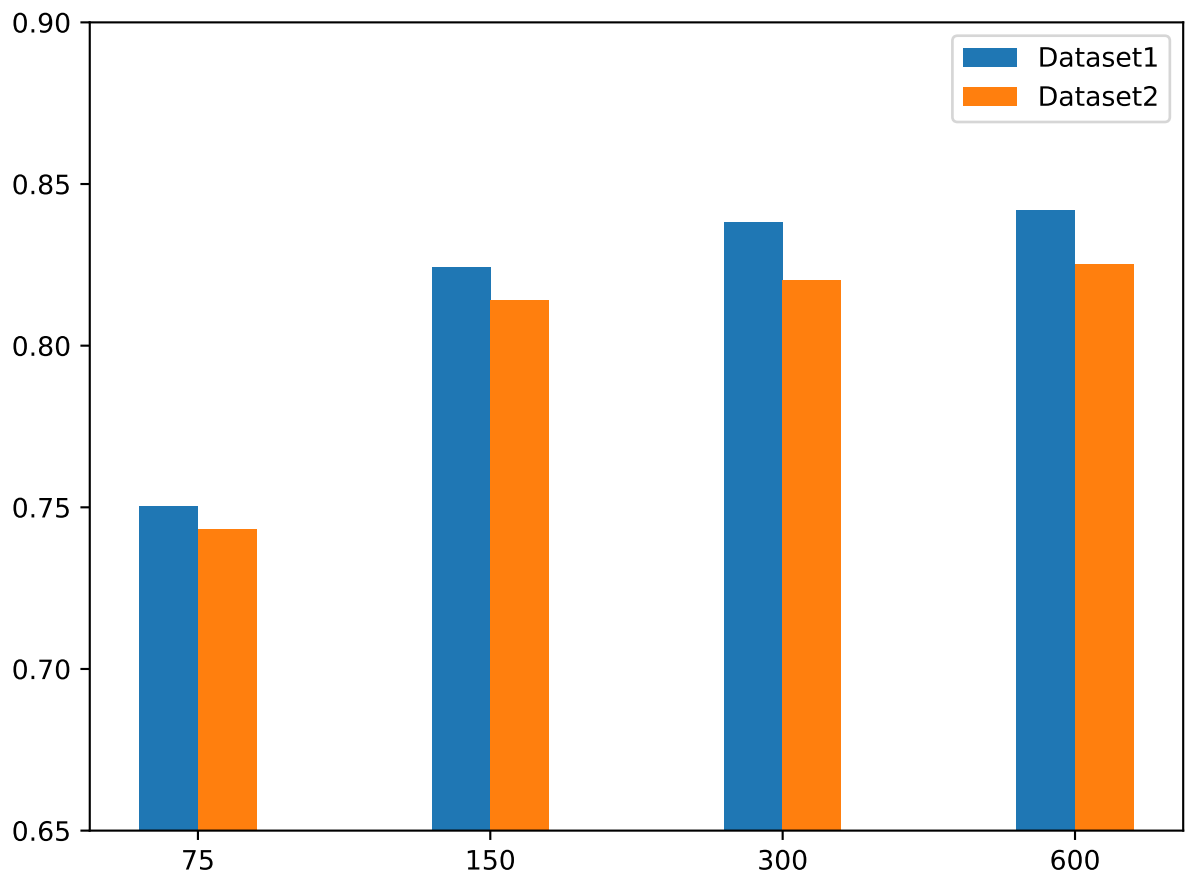

Supplement: Supplementary file 13 — Additional file 13. AUPR at different dimension of embedding vectors of lncRNA [file 12859_2021_4073_MOESM13_ESM.pdf]
