# Supplementary material for: A representation learning model based on variational inference and graph autoencoder for predicting lncRNA-disease associations
Source: BMC Bioinformatics. 2021 Mar 21;22:136. doi: 10.1186/s12859-021-04073-z (PMC7983260; doi:10.1186/s12859-021-04073-z)
Supplement: Supplementary file 4 — Additional file 4. Case study for breast cancer on Dataset2 [file 12859_2021_4073_MOESM4_ESM.pdf]

Table S3: Top 10 predicted lncRNAs associated with breast cancer on Dataset2

Rank	lncRNA name	PMID
1	TUG1	30098551
2	MIAT	29345338
3	WT1-AS	31621381
4	BANCR	29565494
5	HOXA11-AS	28701685
6	GATA3-AS1	26637396
7	JAZF1-AS1	Unconfirmed
8	HAND2-AS1	31683462
9	HAS2-AS1	25081531
10	FAM30A	Unconfirmed
