# Supplementary material for: A representation learning model based on variational inference and graph autoencoder for predicting lncRNA-disease associations
Source: BMC Bioinformatics. 2021 Mar 21;22:136. doi: 10.1186/s12859-021-04073-z (PMC7983260; doi:10.1186/s12859-021-04073-z)
Supplement: Supplementary file 5 — Additional file 5. Case study for colon cancer on Dataset2 [file 12859_2021_4073_MOESM5_ESM.pdf]

Table S4: Top 10 predicted lncRNAs associated with colon cancer on Dataset2

Rank	lncRNA name	PMID
1	UCA1	30652355
2	PVT1	30504754
3	MHRT	Unconfirmed
4	GAS5	27951730
5	NEAT1	31173354
6	CDKN2B-AS1	31729423
7	TUG1	27634385
8	XIST	29679755
9	HOTTIP	29274585
10	KCNQ1OT1	31040703
