# Supplementary material for: A representation learning model based on variational inference and graph autoencoder for predicting lncRNA-disease associations
Source: BMC Bioinformatics. 2021 Mar 21;22:136. doi: 10.1186/s12859-021-04073-z (PMC7983260; doi:10.1186/s12859-021-04073-z)
Supplement: Supplementary file 8 — Additional file 8. Remarks [file 12859_2021_4073_MOESM8_ESM.pdf]

Remarks of A representation learning model based on variational inference and graph autoencoder for predicting lncRNA-disease associations

Zhuangwei Shi[1], Han Zhang[1,*], Chen Jin[2], Xiongwen Quan[1] and Yanbin Yin[3]
[1]College of Artificial Intelligence, Nankai University, Tongyan Road, Tianjin, 300350, China
[2]College of Computer Science, Nankai University, Tongyan Road, Tianjin, 300350, China
[3]Department of Food Science and Technology, University of Nebraska-Lincoln, 1400 R Street, Lincoln, NE 68588, USA
*Correspondence: zhanghan@nankai.edu.cn

Remark of Problem 1

Remark. Variational inference for graph semi-supervised learning adopts the variational distribution $q(y_u|x_v)$ to approximate $p(y_u|y_l, x_v)$, through minimizing the Kullback-Leibler (KL) divergence of these two distributions.

$$\begin{aligned} &\mathrm{KL}[q(y_u|x_v)||p(y_u|y_l, x_v)] \\ =&\mathbb{E}_{q(y_u|x_v)}[\log q(y_u|x_v) - \log p(y_u|y_l, x_v)]. \end{aligned} \tag{S1}$$

Since KL divergence is non-negative, through $p(y_u|y_l) = p(y_l, y_u)/p(y_l)$ and Jenson's inequality, we can obtain

$$\begin{aligned} &\mathbb{E}_{q(y_u|x_v)}[\log q(y_u|x_v) - \log p(y_l, y_u|x_v)] \\ \geq& -\mathbb{E}_{q(y_u|x_v)}[\log p(y_l|x_v)] \geq -\log p(y_l|x_v). \end{aligned} \tag{S2}$$

Here,

$$\mathbb{E}_{q(y_u|x_v)}[\log q(y_u|x_v) - \log p(y_l, y_u|x_v)], \tag{S3}$$

is called evidence lower bound (ELBO). It is bounded thus can be optimized by variational EM (expectation maximization) algorithm (Neal and Hinton, 1998), which executes the following two steps alternately until convergence.

- E-step (inference): Fix $p(y_l, y_u|x_v)$, and train $q(y_u|x_v)$ to optimize Eq. (S3). Usually, $q(y_u|x_v)$ can be computed by sampling from $p(y_l, y_u|x_v)$.
- M-step (learning): Fix $q(y_u|x_v)$, and optimize Eq. (S3) by approximating $p(y_l, y_u|x_v)$ to $q(y_u|x_v)$.

□

Remark of Assumption 1

Remark. Cai et al. (2010) proposed the singular value thresholding (SVT) algorithm to solve matrix completion problem

$$\min_F \ \|F\|_* + \mu\|\mathcal{P}_\Omega(F - Y)\|_F^2.$$

that F is computed through applying singular value decomposition (SVD) to matrix Y, which is commonly implemented through principal component analysis (PCA). Hinton and Salakhutdinov (2006) suggested that autoencoders can be viewed as nonlinear PCA, and Ji et al. (2017); Jing et al. (2020) found that autoencoders are capable of capturing low-rank representation. Both evidences demonstrate that autoencoder with Y as input and F as output can obtain the optimal solution. □

Remark of Definition 1

Remark. Belkin and Niyogi (2002); Ng et al. (2002); Johnson and Zhang (2007) derived that trace($F^T LF$) can be minimized through solving the eigenvalue decomposition problem of normalized Laplacian matrix L, and the k-dimension representation is the eigenvectors of L corresponding to the second to the $(k+1)$-th smallest eigenvalues. Laplacian eigenmap (Belkin and Niyogi, 2002) are representations of nodes on graph obtained through the eigenvalue decomposition of normalized Laplacian matrix L.

Previous research suggests that graph neural networks are significantly correlated to label propagation (Li et al., 2018; Li et al., 2019). Since label propagation leads to manifold regularization problem (Zhou et al., 2004; Wang and Zhang, 2008; Wang et al., 2009), the representations learned by graph neural networks should also follow manifold constraint (Xu et al., 2019; Liu et al., 2019). Hence, the representations learned by graph autoencoders can be viewed as Laplacian eigenmap via deep learning approach. Hence, if there are two graph autoencoders based on the same graph, their learned representations should be similar provided by manifold consistency.

In VGAELDA, since GNNq and GNNp are based on the sharing of the same graph, and learn representations with the same dimension, the output of the hidden layer of GNNp tend to be similar to low-dimension representations learned by GNNq.

Therefore, representation Z and Z' tend to be similar. Minimizing L_m can be viewed as minimizing trace($F^T LF$). Adding this similarity to the penalty of label propagation network, can be viewed as incorporating Laplacian regularized least square method into graph neural networks. Although graph autoencoder GNNp itself can solve Laplacian eigenmap problem, the alternate training of GNNq and GNNp enhances the capability of label propagation procedure, to capture efficient low-dimensional representations from high-dimensional features.

As VGAELDA adds manifold loss without the computation of the quadratic form of normalized Laplacian matrix, the adjacency matrix of the graph G in VGAELDA only need to be estimated sketchily. □

Remark of Definition 2

Remark. We define the co-training loss L_c similar to Han et al. (2019) 's work. According to Assumption 1, autoencoder can be viewed as a nonlinear PCA. So consider the procedure that we obtain the solution of PCA via truncated SVD (Cai et al., 2010), that singular value decomposition could be applied on Y like this:

$$Y_{m\times n} = U_{m\times r}\Sigma_{r\times r}V^T_{r\times n}, \tag{S4}$$

where U and V denote left and right singular vectors respectively, and $\Sigma = \mathrm{diag}\{\sigma_1, \sigma_2, \cdots, \sigma_r\}$, where σ_i denotes the i-th largest singular value of Y, and the dimension of representation is r. Therefore, suppose $\hat{Z}_l = U\sqrt{\Sigma}, \hat{Z}_d^T = \sqrt{\Sigma}V^T$, then $Y = \hat{Z}_l\hat{Z}_d^T$, and $\hat{Z}_l, \hat{Z}_d$ are representations in lncRNA space and disease space that learned through PCA respectively. Hence for VGAELDA, the efficient Z_l and Z_d could also minimize the mean square error between $Z_l Z_d^T$ and Y. □

References

Mikhail Belkin and Partha Niyogi. Laplacian eigenmaps and spectral techniques for embedding and clustering. In *Advances in Neural Information Processing Systems*, volume 15, pages 585–591, 2002.

Jian-Feng Cai, Emmanuel J. Candès, and Zuowei Shen. A singular value thresholding algorithm for matrix completion. *SIAM J. Optimiz.*, 20(4):1956–1982, 2010. URL `https://doi.org/10.1137/080738970`.

Peng Han, Peng Yang, Peilin Zhao, Shuo Shang, Yong Liu, Jiayu Zhou, Xin Gao, and Panos Kalnis. Gcn-mf: Disease-gene association identification by graph convolutional networks and matrix factorization. In *Proceedings of the 25th ACM SIGKDD International Conference on Knowledge Discovery & Data Mining*, volume 25, page 705–713, 2019.

G. E. Hinton and R. R. Salakhutdinov. Reducing the dimensionality of data with neural networks. *Science*, 313(5786):504–507, 2006. URL `https://doi.org/10.1126/science.1127647`.

Pan Ji, Tong Zhang, Hongdong Li, Mathieu Salzmann, and Ian Reid. Deep subspace clustering network. In *Advances in Neural Information Processing Systems*, volume 30, pages 23–32, 2017.

Li Jing, Jure Zbontar, and Yann LeCun. Implicit rank-minimizing autoencoder. In *Advances in Neural Information Processing Systems*, volume 33, pages 1–11, 2020.

Rie Johnson and Tong Zhang. On the effectiveness of laplacian normalization for graph semi-supervised learning. *J. Mach. Learn. Res.*, 8(53):1489–1517, 2007. URL `http://jmlr.org/papers/v8/johnson07a.html`.

Q. Li, X. Wu, H. Liu, X. Zhang, and Z. Guan. Label efficient semi-supervised learning via graph filtering. In *IEEE/CVF Conference on Computer Vision and Pattern Recognition (CVPR)*, pages 9574–9583, 2019.

Qimai Li, Zhichao Han, and Xiao-Ming Wu. Deeper insights into graph convolutional networks for semi-supervised learning. In *Proceedings of the Association for the Advancement of Artificial Intelligence (AAAI)*, 2018.

Qi Liu, Maximilian Nickel, and Douwe Kiela. Hyperbolic graph neural networks. In *Advances in Neural Information Processing Systems*, volume 32, 2019.

R Neal and G Hinton. *A view of the em algorithm that justifies incremental, sparse, and other variants.* Springer, Dordrecht, 1998.

Andrew Y Ng, Michael I Jordan, and Yair Weiss. On spectral clustering: Analysis and an algorithm. In *Advances in Neural Information Processing Systems*, volume 15, pages 849–856, 2002.

F. Wang and C. Zhang. Label propagation through linear neighborhoods. *IEEE Trans. Knowl. Data En.*, 20(1):55–67, 2008.

J. Wang, H. C. Shen, F. Wang, L. Quan, and C. Zhang. Linear neighborhood propagation and its applications. *IEEE Trans. Pattern Anal. Mach. Intell.*, 31(9):1600–1615, 2009. URL `https://doi.org/10.1109/TPAMI.2008.216`.

Bingbing Xu, Huawei Shen, Qi Cao, Keting Cen, and Xueqi Cheng. Graph convolutional networks using heat kernel for semi-supervised learning. In *International Joint Conference on Artificial Intelligence (IJCAI)*, pages 1928–1934, 2019.

Dengyong Zhou, Olivier Bousquet, Thomas N. Lal, Jason Weston, and Bernhard Schölkopf. Learning with local and global consistency. In *Advances in Neural Information Processing Systems*, volume 16, pages 321–328, 2004.
